# Patient and Health Care Facility Factors Associated with Advanced Cervical Lesions in Zambia Over 13-Year Period

**DOI:** 10.21203/rs.3.rs-7510691/v1

**Published:** 2025-10-21

**Authors:** M. Asiedu-Danso, M. Kalima, F. C. Ng’uni, K. Lishimpi, D. A. Larsen, L. Zhang, Amr S. Soliman

**Affiliations:** Syracuse University; Lusaka Provincial Health Office; Lusaka Provincial Health Office; Ministry of Health; Syracuse University; City University of New York School of Medicine; City University of New York School of Medicine

**Keywords:** Cervical cancer screening, Cervical cancer lesions, Zambia, HIV

## Abstract

**Introduction:**

Zambia has one of the highest burdens of cervical cancer worldwide, driven in part by its high HIV prevalence. In response, the country adopted nurse-led “screen and treat” services. Despite these efforts, anecdotal reports from Lusaka Province suggested an increase in advanced precancerous lesions among first-time screened women. This study aimed to examine patient and health care facility-level factors associated with the diagnosis and treatment of advanced cervical precancerous lesions (cervical intraepithelial neoplasia 2 and 3 (CIN2/3)) to inform targeted improvements in screening and referral practices.

**Methods:**

This study included a retrospective cohort of first-time cervical cancer screened women in all six districts of Lusaka Province, Zambia, from 2010 to 2022. Screening data of women aged 18 and older who underwent visual inspection with acetic acid (VIA) screening were included. Patient-level variables analyzed included age, HIV status, VIA results, and treatment plan. Facility characteristics included facility level and district. Bivariate analyses assessed associations between patient/facility characteristics and advanced cervical lesion treatment. Multivariable logistic regression estimated the adjusted odds of presenting with advanced lesions requiring referral.

**Results:**

A total of 6,768 women were diagnosed with advanced lesions eligible for loop electrosurgical excision procedure (LEEP) treatment. Among those, 1,476 (21.8%) received LEEP treatment, and 4,164 (61.5%) were referred due to complications. Between 2017 and 2022, 53.3% more women received treatment than between 2010 and 2016. Compared with HIV-negative women, newly diagnosed women with HIV were 2.09 times more likely to have complicated advanced lesions (p < 0.0001). Compared to rural facilities, urban facilities had 0.1 -fold lower odds of referral (p < 0.0001). Compared with third-Level hospitals, health posts had the highest odds of referrals (3.3, p < 0.0001), followed by health centers (1.7, p <0.0001).

**Conclusion:**

Increases in screening facilities after 2016 led to increased rates of advanced lesion detection. HIV-positive women who were not receiving antiretroviral treatment (ART) were at increased risk of complicated lesions. Finally, lower-level, and rural facilities had higher referral rates for advanced cases. These findings highlight the need to improve provider training and treatment capacity to reduce unnecessary referrals and efficient local management of screened women at rural and lower-level facilities.

## Introduction

Cervical cancer is the fourth most common cancer among women worldwide, with approximately 660,000 new cases and 350,000 deaths in 2022.^1^ In the same year, approximately 94% of the 350,000 cervical cancer deaths occurred in low- and middle-income countries (LMICs), particularly in sub-Saharan Africa. Zambia has the third highest burden of cervical cancer in the world, with an estimated crude incidence rate of 65.5 per 100,000 women and a mortality rate of 43.4 per 100,000 in 2020.^2^ Zambia’s high incidence has been attributed to the high prevalence of HIV;^3–5^ women living with HIV are 4–6 times at risk of developing cervical cancer.^1^ According to the country’s 2019 HIV impact assessment, women between the ages of 15–59 years had a 14.6% prevalence of HIV.^6^ Lusaka Province which is the most populated province within the country has an HIV prevalence of 20.8%, well above the national average.^7^ These high rates highlight the urgent need for effective cervical cancer screening and treatment.

In 2006, Zambia joined the global community to reduce morbidity and mortality from cervical cancer through the Cervical Cancer Prevention Program (CCPPZ).^8^ Under this program, nurses are trained to provide free cervical screening via visual inspection with acetic acid (VIA) and treatment with cryotherapy (ablation) or loop electrosurgical excision procedure (LEEP).^8^ Women with complex cervical cancer lesions are referred to the University Teaching Hospital (UTH) in Lusaka for further evaluation.^5,8^ LEEP treatment was reserved for patients with cervical intraepithelial neoplasia 2 and 3 (CIN2/3). These lesions are considered high-grade squamous intraepithelial lesions (HSILs) or advanced lesions, and if left untreated, they often progress into cervical cancer.^5^ Advanced lesions among first-time screened women may indicate population underscreening and can lead to delayed diagnosis and low survival rates.^9^ Additionally, complicated advanced lesions in first-time screened women may indicate a high prevalence of high-risk HPV types and inadequate screening, and may require more complex and expensive treatment requiring skilled personnel.^9^ Thus, ensuring efficient screening practices and increased public awareness are imperative. In Zambia, information about screening is disseminated through media campaigns and community outreach efforts led by provincial health offices through nurses and peer educators to increase community and women’s awareness and counteract misconceptions and myths.^8^

Observations and clinical impressions of the program managers and screening professionals in Lusaka, Zambia, suggested that the number of advanced precancer lesions detected at screening facilities has increased. This anecdotal evidence highlights the need for a systematic investigation to verify these clinical impressions. If the impression of increasing rates of advanced lesions is correct, then investigation of the patient- and facility-level factors associated with the diagnosis and treatment outcomes of advanced cervical precancer lesions among first-time screened women in Lusaka Province, Zambia, is warranted. Thus, the aim of this study was to describe the patient and facility characteristics linked to the diagnosis and treatment of advanced cervical precancer lesions in Lusaka Province, Zambia, to inform targeted improvements in screening and referral practices.

## Methods

### Study site

Lusaka Province is the most populated province in Zambia with a population of 2.2 million and a population density of 141 persons per square kilometer.^10^ The province consists of six districts, namely; Chilanga, Chongwe, Kafue, Luangwa, Lusaka and Rufunsa.^7^ Lusaka province has a high HIV-positive prevalence of 20.8% among all residents and a prevalence of 22.4% among women.7 The Zambian government remains the predominant health service provider, with 116 health facilities spread across the province.^7^

### Health System and Study Participants

The study population consisted of all women who had visited cervical cancer screening facilities in Lusaka districts for the first time in their lives from 2010–2022. Electronic VIA screening records for all districts were obtained from the Lusaka Province monitoring and evaluation unit of the Lusaka Province Health Office (LPHO).

Health facilities in Zambia are ranked on the basis of the level of skilled personnel, logistics and equipment and the population served.^11^ The government-owned health facilities involved in cervical screening include health posts, health centers, mini-hospitals, first-level hospitals, second-level hospitals, and third-level hospitals. Health posts are in very remote areas and offer basic services.^11^ They are typically run by nurses or community health workers. Health centers rank higher than health posts and are located in rural or urban locations with more services. The health centers serve as satellite clinics to first- or second-level hospitals.^11^ Mini-hospitals offer services provided by both health centers but are regarded as zonal health centers, more advanced than health centers, but lower than first-level hospitals, which are also referred to as district hospitals that offer medical, surgical, obstetric, diagnostic, and clinical services in support of health center referrals. Second-level hospitals, also referred to as provincial or general hospitals, are found at the provincial level.^11^ These hospitals also act as referrals for first-level hospitals in addition to providing technical support and training for health professionals.^11^ Third-level hospitals, otherwise known as specialist or tertiary hospitals, are the highest-level referral hospitals in Zambia. The hospitals have subspecializations in internal medicine, surgery, pediatrics, obstetrics, gynecology, intensive care, psychiatry, training, and research.^11^ All complicated cases not treated at second-level hospitals are referred to third-level hospitals.^11^

### Inclusion and Exclusion Criteria

**Figure 1:**
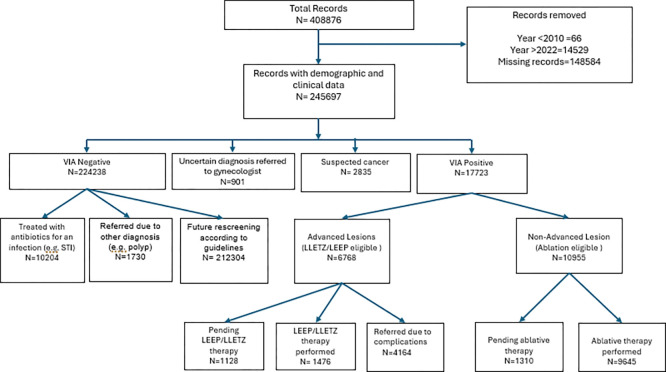
Flow chart of participant inclusion and exclusion for first-time screeners in Lusaka Province screening facilities (2010–2022)

The sources of the data and the inclusion and exclusion criteria are outlined in Fig. 1. Patients included in this study were required to be older than 18 years and screened for cervical lesions between 2010 and 2022 at one of the clinics of the Lusaka Province. This statistical analysis excluded records with incomplete information (n = 3,772,971), patients who did not undergo VIA testing due to contraindications, such as cervical sores (n = 1,312,580), patients under the age of 18 years (n = 8,212), and records before 2010(n = 104,451) or after 2022(n = 14,529).

### Data collection

As part of the standard cervical screening procedures of all the facilities, all patient data were routinely collected and entered on site by trained nurses into electronic databases. In addition to the demographic data of the patients, the data included the patient’s HIV status, VIA results, and treatment or post-screening clinical plan and referrals.

Data queries were adjudicated and de-identified from various electronic spreadsheets maintained by the Monitoring and Evaluation Unit of the Lusaka Provincial Health Office (LPHO). Facility locations and levels were linked to each patient using the health care facility names as the identifiers. The variables included in the final analysis included the woman’s age, HIV status, VIA result, post screening clinical plan, facility name, facility location, facility level, district in which the facility is located, and year of screening.

### Data Management

Data cleaning was conducted in consultation with the CCPPZ Lusaka Province program managers and the coauthors of this manuscript to ensure that lesion types were correctly classified and that health facility names and locations were accurately recorded. Health facility details (name, location, and level) were based on the most recent facility listing provided by the LPHO.^12^ After data cleaning, the facility electronic databases were merged into a single dataset.

Patients screened for cervical lesions had four possible outcomes: VIA negative or positive, uncertain, and suspected cancer. Patients who were negative were scheduled for routine follow-up for 3–5 years depending on their HIV status. Patients with uncertain diagnoses or suspected cancer lesions were referred to gynecologists at higher-level facilities for confirmatory diagnosis and possible treatment. VIA-positive patients received different treatments based on lesion severity. Patients with non-advanced lesions received ablation therapy for precancerous changes. Patients with advanced lesions were scheduled for LEEP or referred if the advanced lesion was complicated.

Age was categorized into six groups: a) patients below the age of 25 (18–24), b) patients between the ages of 25 and 34(25–34), c) patients between the ages of 35 and 44 (35–44), d) patients between the ages of 45 and 54 (45–54), e) patients between the ages of 55 and 64 (55–64), and e) patients 65 and older (≥ 65). HIV screening was often performed if their last HIV test was later than 3 months prior. Thus, HIV status was a four-level categorical variable: HIV negative, HIV positive on antiretroviral medication (ART), Tested HIV positive during screening (Newly Diagnosed), or Refused HIV testing (Unknown). Facility detail groupings were maintained as described in the LPHO registry. Thus, the facility location, type and district were not regrouped. The years of screening were grouped into before and after 2016 (2010–2016 and 2017–2022) on the basis of reported program scale-up and increased funding.^4,13,14^

### Statistical analysis

To describe patient and facility characteristics associated with treatment plans for advanced cervical cancer lesions in Lusaka Province between 2010 and 2022, a chi-square analysis was conducted to examine the proportions of each characteristic and treatment plan (pending LEEP, LEEP performed, or LEEP required but referred). Additionally, a multivariable logistic regression analysis was performed to estimate adjusted odds ratios for the likelihood of presenting with a complicated advanced lesion requiring referral versus an uncomplicated advanced lesion.

The dependent variable was operationalized by considering patients who were booked for LEEP and those who had undergone LEEP as having uncomplicated advanced lesions while those requiring referral were considered complicated advanced lesions. The independent variables considered included, the year period of screening, patient age and HIV status, facility level, location (rural-urban), and district. Independent variables were selected on the basis of their significance from bivariate tests and their significance to the research aim.

### Sensitivity analysis

For the multivariable regression analysis, Rufunsa District was excluded because it had only one reported case of advanced lesion, which led to unstable model estimates. A sensitivity analysis was conducted by combining Rufunsa with the neighboring district of Chongwe, which has similar characteristics; the results remained consistent, yielding the same estimates.

All the statistical tests were two-sided with a significance level of 0.05. Data cleaning and analysis were performed via Excel version 2406, R version 4.4.1 ^15^, and SAS version 9.4.^16^

### Ethics approval and consent to participate:

This secondary data analysis study was conducted in accordance with the ethical principles of the Declaration of Helsinki. Ethical approval was obtained from the University of Zambia Biomedical Research Ethics Committee (ID: 5429–2024) and the Institutional Review Board at Syracuse University (Syracuse, NY, USA; ID: 24–143). As the study utilized only de-identified, routinely collected programmatic data, both institutional review boards waived the requirement for informed consent.

## Results

A total of 6,768 patients were diagnosed with advanced lesions eligible for LEEP treatment (Figure 1). However, only 1,476 (21.9%) patients received LEEP treatment. The majority of patients (61.5%) were referred for complications such as mosaicism present with lesions, and 16.7% of patients were booked for LEEP without any record of completing the treatment (pending LEEP).

Between 2017 and 2022, 53.3% more patients received treatment than between 2010 and 2016 (Table 1). Among those who received treatment, the majority were in the 25–34 years age group (52.6%). This age group also had the highest proportion of patients pending treatment and requiring referrals (41.4% and 45.3%, respectively). Furthermore, patients on ART reported the highest proportion of those receiving treatment and requiring referrals (79.8% and 48.3%, respectively), whereas newly diagnosed patients reported the highest proportion of those pending treatment (35.7%) (Table 1).

The majority of patients were screened in urban facilities across Lusaka Province (96.2%) (Table 1). Most women who were screened for the first time at a first-level hospital received treatment (58.1%), followed by patients who were screened at health centers (35.4%). Interestingly, health centers reported the highest proportion of referrals (41.3%), followed by first-level hospitals (37.2%), third-level hospitals (16.6%) and mini-hospitals (0.1%). Lusaka district had the highest proportion of advanced lesions (92.7%) (Table 1).

The unadjusted odds of a woman being referred due to a complicated lesion was 1.2 times greater between 2017 and 2022 than between 2010 and 2016. After adjusting for patient and facility characteristics, the odds ratio increased to 2.2 (p<0.0001)(Table 2). Age was significantly associated with the odds of being referred for a complicated lesion: however, adjusting for the 55–64 years age group was the only significantly associated age group with 86% greater odds than those with 24 years and younger (p=0.0048). Compared with HIV-negative patients, those newly diagnosed with HIV had 2.1 times greater odds of having complicated advanced lesions, whereas HIV-positive patients on ART had 0.5 times lower odds (p<0.0001) (Table 2).

The adjusted odds ratio of having complicated advanced lesions was 0.1 lower in urban facilities than in rural facilities (p<0.0001) (Table 2). Compared with third-level hospitals, health posts had the highest odds diagnosing complicated advanced lesions (3.3, p<0.0001), followed by health centers (1.7, p<0.0001), and first-level hospitals (1.6, p<0.0001). Second-level hospitals had lower odds compared to third-level hospitals (0.1, p<0.0001). Compared with the district of Lusaka, Kafue district had the highest adjusted odds (2.3, p<0.0001). All other districts reported lower odds (Table 2).

## Discussion

The study identified three key findings. First, the increase in the number of cervical cancer screening facilities in the years 2017 and 2022 resulted in increased rates of diagnosis of advanced cancer lesions. Second, HIV- positive patients are at an increased risk of complicated advanced lesions. Third, health posts and rural facilities are more likely to refer advanced lesions in first time patients screened in Lusaka Province, perhaps indicating that more training is needed for these remote areas.

After 2016, multiple funding initiatives were introduced that supported the cervical cancer screening program leading to an increase in the number of screening facilities across Zambia and particularly several districts within Lusaka Province.^4,13,14^ This facility expansion may have resulted in more women being screened for cervical cancer and thus more advanced lesions being identified.

Our second key finding resonates with previous findings regarding the increased risk of cervical cancer among HIV-positive women and the mitigating effect of ARTs on lesion complications.^17^ The substantial proportion of HIV-positive women with advanced lesions and the greater odds of complicated lesions in newly diagnosed women than in those receiving ART further support this trend. Most funding for cervical cancer screening expansion received after 2016 was aimed at managing HIV/AIDS and its associated conditions. As a result, many cervical cancer screening facilities have been integrated into HIV/AIDS clinics and focused more on screening HIV-positive patients. This may have inadvertently contributed to the high proportion of patients on antiretroviral therapy (ART) accessing screening services for the first time. Integrating cervical cancer screening within HIV care settings offers several advantages, including cost-effectiveness, improved efficiency, and enhanced management of both conditions.^17,18^

Health posts represent the lowest tier of health facilities in Zambia, with the majority of posts located in rural or hard-to-reach areas within the province. Those facilities, which are primarily managed by nurses, often serve as the first point of contact for rural patients seeking cervical cancer screening. However, nurses at these posts may lack the specialized training and logistics required to perform LEEP procedures, particularly in cases involving complications such as mosaicism.^19,20^ As a result, nurses employed at the posts are more likely to refer patients when they feel unqualified or uncomfortable managing such cases, which may partly explain the high referral rates observed from these facilities. This trend has also been observed in previous studies conducted in other LMICs.^21,22^ This tendency is further reinforced by national treatment guidelines, which advise nurses to refer patients who may require LEEP–especially when complications are suspected.^23^ Moreover, because VIA has relatively low specificity, this guidance may inadvertently lead to excessive referrals for lesions that are not cancerous.^21,22,24^ The ongoing policy shift toward the use of Pap smears and HPV/DNA testing, which offers greater diagnostic precision, is anticipated to alleviate some excessive referrals.^5^

High rates of pending LEEP procedures may stem from multiple factors, including patients’ hesitancy particularly in contexts where family or spousal approval is needed before initiating treatment, as reported in other studies.^20^ Logistical constraints or temporary unavailability of skilled personnel example when trained staff are on leave, equipment breakdown, and power cuts.^25^ Although first- and third-level hospitals are higher level facilities than health posts and health centers are, they are often situated within facilities that have well-functioning gynecological units. Even if nurses are unempowered or untrained to initiate treatment independently in advanced-level facilities, they often rely on physicians for input and/or clearance. Consequently, referrals from such lower facilities may reflect systemic limitations in provider training and empowerment rather than a greater burden of complex cases.^22^

This study has several strengths including the use of a large, province-wide sample that spans all districts in Lusaka Province over a 13-year period, enhancing the representativeness and generalizability of the findings within the Luska Province region. By leveraging routinely collected programmatic data, the analysis reflects real-world implementation of cervical cancer screening services, offering insights that are directly applicable to ongoing health system practices. The study’s incorporation of both patient-level characteristics (age and HIV status) and facility-level factors (facility level and location) allows for a comprehensive assessment of the determinants of advanced lesion diagnosis and treatment patterns. These findings have strong policy relevance and applications, particularly in informing training needs for frontline health workers and guiding the continued integration of cervical cancer screening into existing HIV clinics across the province and the country. A limitation of this study is missing data, which is not uncommon in low-income countries. To address this data cleaning was conducted in consultation with program managers. For instance, records containing only screening IDs (CCPID) were determined to be generated by nurses prior to the arrival of patients. As a result, some IDs do not always correspond to patient data. The accuracy and completeness of variables such as HIV status, lesion classification, and treatment plans depend on the quality of nurse-entered records at the point of care.

## Conclusion

This study highlights important insights into the evolving landscape of cervical cancer screening in Lusaka Province. The increase in the number of screening facilities between 2017 and 2022 resulted in a 53% increase in the number of diagnoses of advanced lesions, particularly among HIV-positive patients and rural patients presenting at rural health posts. These trends underscore the impact of integrating cervical cancer screening into HIV clinics, a strategy that has expanded access but also revealed challenges in treatment capacity. The high rate of referrals from health posts and first-level hospitals reflects gaps in provider training and treatment logistics, reinforcing the need for continued investment in health worker training and infrastructure. While the use of routinely collected data inherently presents some limitations, including missing information, the study’s large, province-wide sample size and real-world relevance make it a valuable contribution to cervical cancer prevention efforts in Sub-Saharan Africa. These findings can inform targeted policy and programmatic responses to strengthen screening, referral, and treatment pathways across the continuum of cervical cancer care.

## Figures and Tables

**Table 1:** Patients and facility characteristics for first time screeners by treatment plan in Lusaka Province, 2010–2022

Characteristic	Overall N=6768	Received Treatment (N=1476, 21.81%)	Pending Treatment (N=1128, 16.67%)	Referred For Treatment (N=4164, 61.52%)	p-value
**Year**					
2010–2016	1580 (23.35%)	14 (0.89%)	656 (41.52%)	910 (57.59%)	**<.0001**
2017–2022	5188 (76.65%)	1462 (28.18%)	472 (9.10%)	3254 (62.72%)	
**Age**					**<.0001**
≤24	435 (6.43%)	101 (6.84%)	71 (6.29%)	263 (6.32%)	
25–34	3130 (46.25%)	776 (52.57%)	467 (41.40%)	1887 (45.32%)	
35–44	2232 (32.98%)	422 (28.59%)	411 (36.44%)	1399 (33.60%)	
45–54	768 (11.35%)	156 (10.57%)	133 (11.79%)	479 (11.50%)	
55–64	139 (2.05%)	14 (0.95%)	26 (2.30%)	99 (2.38%)	
≥65	64 (0.95%)	7 (0.47%)	20 (1.77%)	37 9 (0.89%)	
**HIV**					
Negative	1319 (19.49%)	263 (17.82%)	249 (22.07%)	807 (19.38%)	**<.0001**
Positive					
On ART	3542 (52.33%)	1178 (79.81%)	355 (31.47%)	2009 (48.25%)	
Newly Diagnosed	1604 (23.70%)	28 (1.90%)	403 (35.73%)	1173 (28.17%)	
Refused Testing (Unknown)	303 (4.48%)	7 (0.47%)	121 (10.73%)	175 (4.20%)	
**Facility Location**					**<.0001**
Rural	255 (3.77%)	31 (2.10%)	9 (0.80%)	215 (5.16%)	
Urban	6513 (96.23%)	1445 (97.90%)	1119 (99.20%)	3949 (94.84%)	
**Facility Level**					**<.0001**
Third Level hospital	1244 (18.38%)	28 (1.90%)	527 (46.72%)	689 (16.55%)	
Second Level hospital	38 (0.56%)	24 (1.63%)	7 (0.62%)	7 (0.17%)	
First Level hospital	2602 (38.45%)	858 (58.13%)	194 (17.20%)	1550 (37.22%)	
Mini Hospital	8 (0.12%)	1 (0.07%)	3 (0.27%)	4 (0.10%)	
Health Center	2615 (38.64%)	522 (35.37%)	372 (32.98%)	1721 (41.33%)	
Health Post	261 (3.86%)	43 (2.91%)	25 (2.22%)	193 (4.63%)	
**Facility District**					**<.0001**
Chilanga	51 (0.75%)	10 (0.68%)	7 (0.62%)	34 (0.82%)	
Chongwe	237 (3.50%)	55 (3.73%)	9 (0.80%)	173 (4.15%)	
Kafue	171 (2.53%)	41 (2.78%)	19 (1.68%)	111 (2.67%)	
Luangwa	32 (0.47%)	3 (0.20%)	2 (0.18%)	27 (0.65%)	
Lusaka	6275 (92.72%)	1367 (92.62%)	1091 (96.72%)	3817 (91.67%)	
Rufunsa	2 (0.03%)	0 (0.00%)	0 (0.00%)	2 (0.05%)	

**Table 2:** Unadjusted and adjusted odds ratios for patients and facility factors associated with treatment (LEEP) versus referral among first time screeners in Lusaka Province (2010–2022)

Characteristic	Crude Model			Adjusted Model		
	Odds Ratio	95% CI	p-value	Odds Ratio	95% CI	p-value
**Year**						
2010–2016	**Ref.**			**Ref.**		
2017–2022	1.24	1.11–1.39	0.0002	2.18	1.84–2.59	<.0001
**Age**						
≤24	**Ref.**			**Ref.**		
25–34	0.99	0.81–1.22	0.9452	1.01	0.81–1.25	0.9414
35–44	1.10	0.89–1.36	0.3823	1.15	0.92–1.44	0.2143
45–54	1.08	0.85–1.38	0.5127	1.14	0.89–1.47	0.3089
55–64	1.62	1.07–2.45	0.0228	1.86	1.21–2.87	0.0048
≥65	0.90	0.53–1.53	0.6864	1.04	0.59–1.84	0.8835
**HIV**						
Negative	**Ref.**			**Ref.**		
Positive						
On ART	0.83	0.73–0.95	0.0051	0.53	0.45–0.61	<.0001
Newly Diagnosed	1.73	1.48–2.02	<.0001	2.09	1.76–2.49	<.0001
Refused Testing (Unknown)	0.87	0.67–1.12	0.2713	1.33	1.01–1.75	0.0442
**Facility Location**						
Rural	Ref.			Ref.		
Urban	0.29	0.20–0.40	<.0001	0.09	0.05–0.19	<.0001
**Facility Level**						
Third Level hospital	**Ref.**			**Ref.**		
Second Level hospital	0.18	0.08–0.42	<.0001	0.10	0.04–0.26	<.0001
First Level hospital	1.19	1.04–1.36	0.0139	1.63	1.38–1.93	<.0001
Mini Hospital	0.81	0.20–3.24	0.7605	0.88	0.19–4.14	0.8689
Health Center	1.55	1.35–1.78	<.0001	1.68	1.42–2.00	<.0001
Health Post	2.29	1.70–3.08	<.0001	3.29	2.40–4.50	<.0001
**Facility District**						
Lusaka	**Ref.**			**Ref.**		
Chilanga	1.29	0.72–2.31	0.3961	0.13	0.05–0.34	<.0001
Chongwe	1.74	1.30–2.33	0.0002	0.35	0.21–0.58	<.0001
Kafue	1.19	0.87–1.64	0.2808	2.31	1.51–3.54	0.0001
Luangwa	3.48	1.34–9.04	0.0106	0.18	0.05–0.60	0.0054

## Data Availability

The datasets supporting the conclusions of this study are available from the Monitoring and Evaluation Unit of the Lusaka Provincial Health Office (LPHO) under the auspices of the Ministry of Health of Zambia. Data are, however, available from the authors upon reasonable request and with permission of the Lusaka Provincial Health Office (LPHO).

## References

[1] WHO. WHO Fact Sheet: Cervical cancer. November 17. 2023. Accessed February 7, 2024. https://www.who.int/news-room/fact-sheets/detail/cervical-cancer

[2] WHO. Zambia steps up cervical cancer screening with HPV testing. WHO | Regional Office for Africa. February 2. 2024. Accessed June 8, 2024. https://www.afro.who.int/countries/zambia/news/zambia-steps-cervical-cancer-screening-hpv-testing

[3] WHO, Zambia, Cervical Cancer Profile. Published online 2021. Accessed February 7, 2024. https://cdn.who.int/media/docs/default-source/country-profiles/cervical-cancer/cervical-cancer-zmb-2021-country-profile-en.pdf?sfvrsn=febb231e_38&download=true

[4] Tsehaiu M. February. Global Fund support for screening and treatment of cervical cancer in Zambia. Presented at: 2020. https://togetherforhealth.org/wp-content/uploads/Global-Fund-support-to-cervical-cancer-programming_revised.pdf#:~:text=Zambia%20was%20among%20the%208%20PRRR%20supported,vaccine%20targeting%20young%20girls%209%2D14%

[5] Ministry of Health, Zambia. Zambia National Guidelines For Cervical Cancer Screening. Published online October; 2023.

[6] ZAMPHIA, ZAMBIA POPULATION-BASED HIV IMPACT ASSESSMENT, ZAMPHIA. 2016. Published online 2019. https://phia.icap.columbia.edu/wp-content/uploads/2019/02/ZAMPHIA_Summary_Sheet_Final.pdf

[7] Zambia Statistics Agency. 2010 Census of Population and Housing: Lusaka Province Analytical Report. Published online March 2014.

[8] Nyambe A, Kampen JK, Baboo SK, Van Hal G. The impact of the social environment on Zambian cervical cancer prevention practices. BMC Cancer. 2018;18:1242. 10.1186/s12885-018-5164-1. 30541491 PMC6292082

[9] WHO. Diagnosis and treatment of invasive cervical cancer. In: Comprehensive Cervical Cancer Control: A Guide to Essential Practice. 2nd Edition. World Health Organization. 2014. Accessed July 7, 2025. https://www.ncbi.nlm.nih.gov/books/NBK269603/25642554

[10] Zambia Statistics Agency, Census National Analytical Report. 2022. Published online 2025. https://www.zamstats.gov.zm/wp-content/uploads/2025/07/2022-Census-National-Analytical-Report.pdf

[11] Ministry of Health, Zambia. Zambia Health Facility Registry. Published online October; 2023.

[12] Lusaka Provincial Health Office. Annual Statistical Report 2023. Published online 2024.

[13] Parham GP, Mwanahamuntu MH, Kapambwe S, Population-Level Scale-Up of Cervical Cancer Prevention Services in a Low-Resource Setting: Development, Implementation, and Evaluation of the Cervical Cancer Prevention Program in Zambia. PLoS ONE. 2015;10(4):e0122169. 10.1371/journal.pone.0122169. 25885821 PMC4401717

[14] WHO. WHO Country Office signs a financing agreement with African Development Bank (ADB) on leveraging mobile technology to improve access to basic health services | WHO | Regional Office for Africa. July 4. 2025. Accessed July 5, 2025. https://www.afro.who.int/news/who-country-office-signs-financing-agreement-african-development-bank-adb-leveraging-mobile

[15] R Core Development Team. R: A Language and Environment for Statistical Computing. HttpwwwR-Proj. Published online 2010.

[16] SAS Institute Inc. SAS Software.

[17] Pavone G, Marino A, Fisicaro V, Entangled Connections: HIV and HPV Interplay in Cervical Cancer-A Comprehensive Review. Int J Mol Sci. 2024;25(19):10358. 10.3390/ijms251910358. 39408687 PMC11477307

[18] Plotkin M, Besana GV, Yuma S, Integrating HIV testing into cervical cancer screening in Tanzania: an analysis of routine service delivery statistics. BMC Womens Health. 2014;14(1):120. 10.1186/1472-6874-14-120. 25271025 PMC4190378

[19] Lavelle AE, Su D, Kahesa C, Soliman AS. Needs for Professional Education to Optimize Cervical Cancer Screenings in Low-Income Countries: a Case Study from Tanzania. J Cancer Educ Published online September 11, 2017:1–6. 10.1007/s13187-017-1276-6 PMC5845764 28895070

[20] Anaman-Torgbor J, Angmorterh SK, Dordunoo D, Ofori EK. Cervical cancer screening behaviours and challenges: a sub-Saharan Africa perspective. Pan Afr Med J. 2020;36:97. 10.11604/pamj.2020.36.97.19071. 32774656 PMC7392861

[21] Bhatla N, Mukhopadhyay A, Joshi S, Visual inspection for cervical cancer screening: evaluation by doctor versus paramedical worker. Indian J Cancer. 2004;41(1):32–6. 15105577

[22] Sherigar B, Dalal A, Durdi G, Pujar Y, Dhumale H. Cervical cancer screening by visual inspection with acetic acid-interobserver variability between nurse and physician. Asian Pac J Cancer Prev. 2010;11(3):619–22. 21039026

[23] Ministry of Health, Zambia. CERVICAL CANCER ELIMINATION PROGRAM LEEP/LLETZ CLINICAL SKILLS COURSE MANUAL. Published online 2023. https://www.moh.gov.zm/wp-content/uploads/filebase/guidelines/LEEP-Manual-2023.pdf

[24] Mustafa RA, Santesso N, Khatib R, Systematic reviews and meta-analyses of the accuracy of HPV tests, visual inspection with acetic acid, cytology, and colposcopy. Int J Gynaecol Obstet Off Organ Int Fed Gynaecol Obstet. 2016;132(3):259–65. 10.1016/j.ijgo.2015.07.024. 26851054

[25] Irowa O, Adeyeye OS, Ujah OI, Ogwuche PE, Otache AE, Agulebe CJ. Assessment of barriers to cervical cancer screening at primary health care centers in Makurdi, North-Central Nigeria: a mixed-methods study. BMC Cancer. 2025;25:1099. 10.1186/s12885-025-14494-1. 40598064 PMC12211423

